# Complex trade-offs in a dual-target visual search task are indexed by lateralised ERP components

**DOI:** 10.1038/s41598-024-72811-3

**Published:** 2024-10-01

**Authors:** Dion T. Henare, Jan Tünnermann, Ilja Wagner, Alexander C. Schütz, Anna Schubö

**Affiliations:** 1 Auckland University of Technology, 90 Akoranga Drive, Auckland, 0627 New Zealand; 2 Philipps-University of Marburg, Marburg, Germany; 3 Justus Liebig University, Giessen, Germany

**Keywords:** Attention, Human behaviour

## Abstract

In everyday tasks, the choices we make incorporate complex trade-offs between conflicting factors that affect how we will achieve our goals. Previous experimental research has used dual-target visual search to determine how people flexibly adjust their behaviour and make choices that optimise their decisions. In this experiment, we leveraged a visual search task that incorporates complex trade-offs, and electroencephalography (EEG), to understand how neural mechanisms of selective attention contribute to choice behaviour in these tasks. On each trial, participants could choose to respond to the gap location on either of two possible targets. Each target was colour coded such that colour indicated which of the two had the easier gap discrimination. Orthogonally, we manipulated the set size of coloured distractors to modulate how efficiently each target could be found. As a result, optimised task performance required participants to trade-off conflicts between the ease of finding a target given the current set size, and the ease of making its associated gap discrimination. Our results confirm that participants are able to flexibly adjust their behaviour, and trade-off these two factors to maintain their response speed and accuracy. Additionally, the N2pc and SPCN components elicited by search displays could reliably predict the choice that participants would ultimately make on a given trial. These results suggest that initial attentional processes may help to determine the choice participants make, highlighting the central role that attention may play in optimising performance on complex tasks.

## Introduction

In real-world situations, high-level goals like finding something to eat, or searching for a space in a busy car park have many different possible solutions. At any given moment, our decision about where to park our car must quickly trade off a range of factors like how far it is from our current location, the ease of manoeuvring into the space, and its distance from the store. While complex decisions like this are present in everyday life, experimental tasks incorporating open-ended trade-offs with multiple targets are a relatively recent development. These tasks have been critical for establishing the way that people can flexibly adjust their behaviour from moment to moment in order to maximise value^1,2^ and minimize effort^3–5^ within a context.

Navalpakkam and colleagues^1^ evaluated the trade-off between target salience and reward value by developing a search task with two possible targets on each display. Participants could select either the horizontal or vertical target each worth a different number of points depending on block, while six line distractors were used to modulate the salience of the targets on a given trial. Their results showed that participants were able to dynamically trade off the saliency of a target and its reward value to maximise their gains across the task. A similar effect was observed by Irons and Leber^4,5^ who used a task in which participants chose to respond to either a red or blue target on each trial. Irons and Leber manipulated the color of surrounding distractors, shifting the hue trialwise from red to blue and back again. Their results showed that participants can adapt their target choice based on the effective distractor set size, opting for the blue target when there are a small number of blue items to search through, and switching to the red target when there are a small number of red items.

The results of Irons and Leber^4^ in particular highlighted how this target switching would trade off against effort minimisation. As a result of the way that the distractor context changed predictably and gradually across trials, participants moved from contexts dominated by one colour, through contexts in which it was balanced, to contexts dominated by the opposing colour. Results showed that rather than switching target as soon as the displays moved past the balanced point, participants delayed their decision to switch. This likely reflects the cognitive effort required to switch task sets that has been observed in other voluntary task switching tasks^6–8^. Effort minimisation incentivised participants to stay focused on the current target choice for some time, even after it became the less salient of the two options. This has been confirmed more recently by Bergmann et al.^3^ who systematically manipulated the number of distractors in each target colour using a task where the colour itself was task-irrelevant. Again, their results showed that participants can flexibly adapt their response to account for distractor set size, but that these decisions involve a trade off against the effort involved in switching.

The trade-off between search costs and perceptual performance was investigated in our recent dual-target visual search paradigm.^2^ In that paradigm, participants had to search for one of two available targets, on either of which they had to report the location of a gap. To manipulate perceptual performance, the gap was large in the easy target and small in the difficult target. Both targets were surrounded by a coloured circle that indicated the difficulty of the target. To manipulate the search costs for the easy and difficult target, the relative number of “easy” and “difficult” coloured distractors was varied across trials. Both, the perceptual performance and search costs were reflected in fixations and target choices in this task. A comparison with an optimal observer model suggested that fixations and choices were nearly optimal, given noise in the representation of the targets’ value and noise in the selection of fixation locations. While these results demonstrate that humans easily perform complex trade-offs to optimize performance in such tasks, they do not reveal the underlying neural processes.

Visual selective attention is likely critical to this type of task optimization given its central role in determining the priority of visual information. Attention selects information based on a combination of top-down mechanisms guided by a person’s conscious goals, bottom-up information based on the physical stimulus attributes, and selection history which incorporates a person’s previous selection experience^9,10^. The Posner cuing task^11^, for example, demonstrates the way that top-down knowledge of a target’s likely location speeds behavioural responses to the target, whereas the additional singleton task^12^ has been used to demonstrate the way that distractors that capture bottom-up attention can divert it from a top-down goal. Recent work evaluating the role of selection history has shown how it similarly drives attention guidance despite the top-down preferences of a person, but can also be used to suppress the interference caused by an otherwise salient distractor^13–17^. However, while extensive work has shown how these attention mechanisms impact on selection of a single target object, relatively little work has attempted to systematically evaluate how attentional selection contributes to complex decisions in a more open, multi-target task.

While attention is intrinsically involved in determining which items will be searched in what order, an open question remains as to whether it determines the choice a person ultimately makes. Milosavljevic and colleagues^18^ attempted to investigate how initial attention priority may impact on downstream processing of an object using displays of food items. In their task, they manipulated the initial brightness of one item in the display to manipulate attentional priority of that item through bottom-up processing. Their results showed that in a simple choice between two items, the salient item was more likely to be chosen for fixation by participants, suggesting that choice was partly driven by initial attention priority. Similar studies have used the same logic of manipulating factors like colour, size, visual clutter, and position to demonstrate how these bottom-up processes can drive participant choice. As a result, evidence accumulation models of decision-making assume that attention plays a central and active role in decisions. In the attentional drift diffusion model,^19^ for example, attending to an item causes an accumulation of evidence in favour of choosing that target.

On the other hand, it need not be the case that attention plays such a central and active role in decision-making. In rational and bounded rational models of decision-making, attention is assumed to take a relatively passive role, extracting all relevant information from a display but not informing which item a person should choose.^20^ Most studies described earlier that demonstrated how salience drives choice use eye-movements as a measure of item choice. In everyday life, however, a person fixates many more items than they can ultimately choose to interact with. Navalpakkam and colleagues,^21^ manipulated salience of items on a web page to test how this separately impacted both eye movements and final choice (in the form of a mouse click). Their findings showed that salience affects both eye movements and choice, however, its effect on choice was much smaller and choice was more likely to be driven by higher-level properties like preference. In addition, work using salient distractors to capture attention frequently report that this can slow participants, but has minimal impact on the accuracy of their responses. In a study that flashed salient banners during a decision-making task, Day and colleagues^22^ reported that this impacted on eye-movements in the task but, again, did not alter the manual responses participants ultimately made. Taken together the results are inconsistent about whether attention drives downstream choice behaviour. One limitation of this work is the reliance on salience as a way to infer attentional priority of an item, rather than measuring the initial attentional priority of that item directly on each trial.

Electroencephalography (EEG) provides an ideal way to measure the ongoing dynamics of brain processing, and dissociate attentional selection from choice responses. In particular, research on attention has made use of a set of lateralised event-related potential (ERP) components to observe the dynamics of early visual attention. N2pc is one prominent lateralised component of the event-related potential that occurs approximately 200 ms post-stimulus, and is measured as an increased negativity at posterior electrodes contralateral to a task-related stimulus^23,24^. This component reflects the distribution of spatial attention to a relevant stimulus, indexing selection of objects in the contralateral visual field^25–27^. The amplitude of N2pc has been shown to modulate as a function of the relevance of a stimulus to top-down feature-based goals^28–31^ as well as implicit biases developed through selection history^8,13,32–37^. When the selected targets require maintenance within working memory for short periods of time before a choice can be made, we also see a sustained posterior contralateral negativity following N2pc (beginning approximately 400 ms post-stimulus)^38–40^. The amplitude of SPCN is determined primarily by the amount of information stored within working memory and, therefore, has been associated with working memory storage and capacity^40,41^. Given their reflection of target selection and maintenance, these components are ideal candidates for evaluating how early attention processes may determine choice behaviour.

Additionally, contralateral positivities are frequently observed in attention tasks, however, their timing and functional significance are less concrete. While later positivities (approximately 350 ms post-stimulus) are typically associated with distractor suppression and disengagement, early lateralised positivities (approximately 100 ms post-stimulus) have been suggested to reflect sensory imbalance of a display^23,42^, intertrial priming biases^43^, and proactive suppression^13^. Taken together, this set of lateralised ERP components provide a read out of the set of attentional processes that precede a behavioural response. However, the vast majority of time these components are measured using tasks with a single pre-defined target and, therefore, their contribution to choice in a more open-ended task design remains unknown.

To this end, we used a dual-target visual search paradigm where participants could respond to either of two competing target rectangles^2^. For one of these targets, the discrimination required to make a response was relatively easy while for the other target, this discrimination was more difficult. Additionally, each target was surrounded by a different coloured ring, which indicated the target gap’s discriminability (e.g., red contains the easy target, blue contains the difficult target). Orthogonal to this factor we manipulated distractor set size, that is, the number of non-target rectangles that were surrounded by a ring matching each target colour. By modulating the number of distractors matching each target, participants were forced to trade off the search costs involved in finding a target with the difficulty of responding to that target given its associated gap discriminability. Based on our previous findings outlined above^2^ we expect that participants should successfully trade-off search costs and gap discriminability across conditions. Additionally, if initial attentional selection contributes to the downstream choice that a person makes in our task, the N2pc, as an index of target selection, should be predictive of target choice. Alternatively, if attention’s role is to passively acquire relevant information (as assumed by rational, and bounded rationality models of decision-making), N2pc amplitude across conditions may predict the speed of responses, but will not be related to the choice that participants ultimately make.

## Methods

### Participants

In total, 31 participants were recruited to participate in the experiment. Six participants could not perform the task accurately in the first session; these participants were not invited back for the EEG session and their data were not used. Of the 25 participants who completed both sessions, 18 were female, and all were right-handed. For the ERP analyses, 18 participants were included in the final analyses after EEG pre-processing removed participants with a large number of noisy trials (see *EEG processing* for details). Ages ranged from 19 to 24, and the mean age was 21.00 (SD = 1.73). With regard to the sample size, note that we employed Bayesian parameter estimation and model comparison as an inferential framework (which quantify evidence in the data for the models), and for which a-priori power analysis is not obligatory as in null-hypothesis significance testing (which is centred on controlling long-term error rates). Nevertheless, we aimed to obtain a similar sample size as earlier studies had used (see Abbasi et al., 2023; Henare & Schubö, 2021; Wagner et al., 2023), to maximize the chance of obtaining informative results. All procedure were conducted in accordance with the ethical guidelines laid down in the 1964 Declaration of Helsinki and was approved by the ethics committee of the Marburg University, Department of Psychology. All participants provided informed consent prior to engaging in the study, including consent for their data to be published in a scientific article.

### Stimuli

Displays consisted of 16 rings, which each had a diameter of 1.38 ${}^{\circ }$ of visual angle and a line thickness of 0.09 ${}^{\circ }$. Within each ring was a rectangle of 0.74 ${}^{\circ }$ × 0.29 ${}^{\circ }$ with a line thickness of 0.06 ${}^{\circ }$. Each rectangle had a gap in the centre of one of its long sides, that varied in size based on target type and participant according to the staircase procedure outlined below. A fixation cross was maintained throughout the experiment (0.57 ${}^{\circ }$ × 0.57 ${}^{\circ }$). The display background was a dark grey (RGB:60,60,60), while the fixation cross and non-coloured rings were all a light grey (RGB:158,158,158), and the rectangles were a medium grey (RGB:109,109,109). Coloured rings were either red (RGB:225,132,124) or blue (RGB:102,161,229). The ring colours (light grey, red, and blue) were balanced in terms of their physical luminance using a luminance meter (Konica Minolta LS-100).

### Experimental procedure

Participants attended two separate sessions that were no more than two days apart. In the first session, behavioural tasks were used to calibrate the two difficulty levels that were required for day two, and fixation control was implemented using an eye-tracker to train participants to maintain fixation. In order to attain a difficult and an easy gap size for each person, a staircase procedure was used to find the gap sizes that resulted in 70% accuracy and 90% accuracy respectively. In the second session, participants were set up for EEG recording and repeated a short version of the staircase procedure to help both remind them of the task, and to remove any practice effects. Participants then started the EEG task. This began with a short practice period in which participants completed blocks of trials until their average accuracy on the most recent block exceeded 70%. They then moved on to the full experiment which ran for 30 min (excluding breaks). Participants were rewarded with one point for each correct answer and were encouraged to perform the tasks as quickly and accurately as possible in order to maximise their earnings within the time limit. Each block contained 56 trials, and participants completed an average of 890 trials (se = 4.75) within the 30-min time limit, resulting in an average of approximately 127 trials per set-size condition.

### Experimental tasks

Before the main EEG task, we conducted a two-stage training with the goal to familiarize the participants with the discrimination and to determine individual easy (ca. 90% correct) and difficult (ca. 70% correct) gap sizes for the Landolt-like Cs that were used for the discrimination component of the task. These pre-experimental tasks are described in Supplementary Information S1 and S2.

In the main EEG task, each trial began with a 500–1000 ms fixation followed by a 200 ms search display, and then a fixation display lasting until response (up to a maximum of 1500 ms). The search display consisted of 16 rings, eight rings to the left of fixation and eight rings to the right of fixation. The rings on each side were arranged in two columns of four, and the layout of each column was curved to ensure that rings within a column were equidistant from fixation. In this way, stimuli can be thought of as falling along the imaginary line of either an inner or outer circle around fixation with a radius of 6 and 9 degrees of eccentricity respectively. Each ring contained a rounded rectangle with a gap in the centre of one of the long sides. One of these rectangles was oriented horizontally, one of these rectangles was oriented vertically, and the remaining 14 rectangles were tilted at a 45-degree angle. Participants were required to select either the vertical or horizontal rectangle, and indicate the edge that contained a gap. Responses were made on an ergodex keypad using keys arranged like the arrow keys of a standard computer keyboard. The two target locations were selected randomly on each trial with two restrictions. Firstly, that they were always presented on opposite sides of fixation, one left and one right, and secondly that they would always be the same distance from fixation i.e. both targets would be presented on the inner circle of rings, or both on the outer circle.

Two independent factors were manipulated in order to establish a trade-off. Firstly, the two targets differed with regard to the difficulty of the gap judgement. One rectangle contained a smaller gap size that was calibrated to be reportable on approximately 70% of trials, whereas the other rectangle contained a larger gap size that was calibrated to be reportable on approximately 90% of trials.

Secondly, possible target locations were highlighted by colouring a portion of the rings either red or blue, with the ratio of red to blue rings manipulated across trials. Locations of the coloured rings were randomly determined on each trial, with the restriction that each colour was constrained to one side of fixation (all red rings on one side, all blue rings on the other). In total, there were always eight coloured rings on screen, therefore, the ratio of red to blue rings varied across trials from 1:7 to 7:1 (with an even distribution of every possible ratio). For each participant, one of the colours highlighted the possible locations of the easy target, and the other colour highlighted the possible locations of the difficult target. As a result, the set size for a given difficulty varied as the proportion of red to blue rings varied. If red is associated with the easy judgement, then on trials with one red and 7 blue rings, participants could benefit from directing attention to the easy target immediately and responding. On the other hand, if there were seven red rings and only one blue, participants would have to decide whether to allocate their attention immediately to the difficult rectangle and attempt a judgment, or whether they would prefer to search through the seven red rings to find the easy rectangle and make their judgement.

After each correct response, participants were given feedback indicating that they had gained a reward, one ‘point’, that would be converted to money at the end of the task. The task was run for 30 min (excluding breaks between blocks), and participants were therefore encouraged to perform as many accurate trials as possible within that 30 min in order to maximise their reward at the end of the task.

### EEG recording

EEG activity was recorded continuously at 1000 Hz using Brain Products 64 channel actiCAP Ag/AgCl electodes. The electrodes were arranged according to the modified combinatorial nomenclature (MCN) for the 10–10 system. The online reference was FCz and the COM sensor was located on the z axis between FCz and Cz. EEG activity was preamplified by active electrodes and then passed to a brain products brainamp amplifier with 16 bit A/D conversion, an input impedance of 10 MOhms, and an antialiasing filter with a 1000 Hz low pass cut off. Impedances were kept below 25 kOHms and active shielding was used throughout for attenuating common mode noise.

### EEG processing

EEG data were processed using the EEGLAB toolbox in MATLAB. First, data were down sampled to 500 Hz, and filtered with a high-pass FIR filter (pop_eegfiltnew) with a passband of 0.1 Hz. Automatic subspace reconstruction was used to clean raw data and to identify any bad channels (which were then reconstructed using spherical interpolation). 700 ms segments were created around the onset of the search display (-200 to 500 ms) excluding trials with an incorrect response, and baselined so that the average of the 200 ms prestimulus period was zero. We then re-referenced the data to linked mastoids and applied a low-pass FIR filter with a passband of 35 Hz. In order to identify eye movements a hEOG channel was created by taking the difference between the two channels located on the outer canthi of each eye, and a vEOG channel was created by taking the difference between electrodes placed above and below the right eye. Segments containing a horizontal eye-movement (± 35 $\mu$ V in the hEOG) within the first 300 ms post-stimulus, and vertical eye movements (± 80 $\mu$ V in the vEOG) within the first 300 ms were removed. We also removed any trial that contained activity greater than ± 100 $\mu$ V in our electrodes of interest (PO3/4, PO7/8, P3/4, P5/6). Data were pooled by averaging left (PO3/PO7,P3,P5) and right (PO4,PO8,P4,P6) channel clusters and then recategorised as either contralateral or ipsilateral to the visual field containing the easy target. At this stage, if any participant had less than 600 trials remaining then they were removed from further analysis (resulting in 6 exclusions). 18 people therefore remained in the final ERP analyses with trial numbers between 622 and 900 ($M$= 724.06, $SD$ = 64.19). In order to select time windows for the ERP components of interest, a grand average lateralised ERP (contralateral—ipsilateral amplitude) was calculated for all participants and all conditions. The N2pc peak was defined as the peak negativity occurring between 180 and 350 ms, and a 50 ms window was selected around this peak time resulting in a window from 219 to 269 ms post-stimulus. The P1pc peak was defined as the peak positivity occurring within the first 200 ms, and a 50 ms window was selected around this peak time resulting in a window from 103 to 153. Given the sustained nature of SPCN, a wider window was used, extending from 300 ms post-stimulus until the end of the epoch.

### Statistical analysis

Throughout the results, the independent variable of interest was number of easy coloured rings. This varied across the levels 1, 2, 3, 4, 5, 6 7. We implemented Bayesian generalized linear mixed models using the Bambi library^44^ and PyMC^45^, utilising the NUTS sampler^46^ to draw 6000 posterior samples (after 4000 tuning samples). Dependent measures included target preference and response time. As predictors we used the easy target set size, and for some model variants we included different lateralised ERP component amplitudes (N2pc and SPCN). To investigate the impact of the processes associated with these components on target (set) selection, we compared different variants in a formal model comparison using Pareto-smoothed importance sampling leave-one-out (LOO) cross validation (Vetari et al., 2017) .

## Results

### Behavioural results

**Fig. 1:**
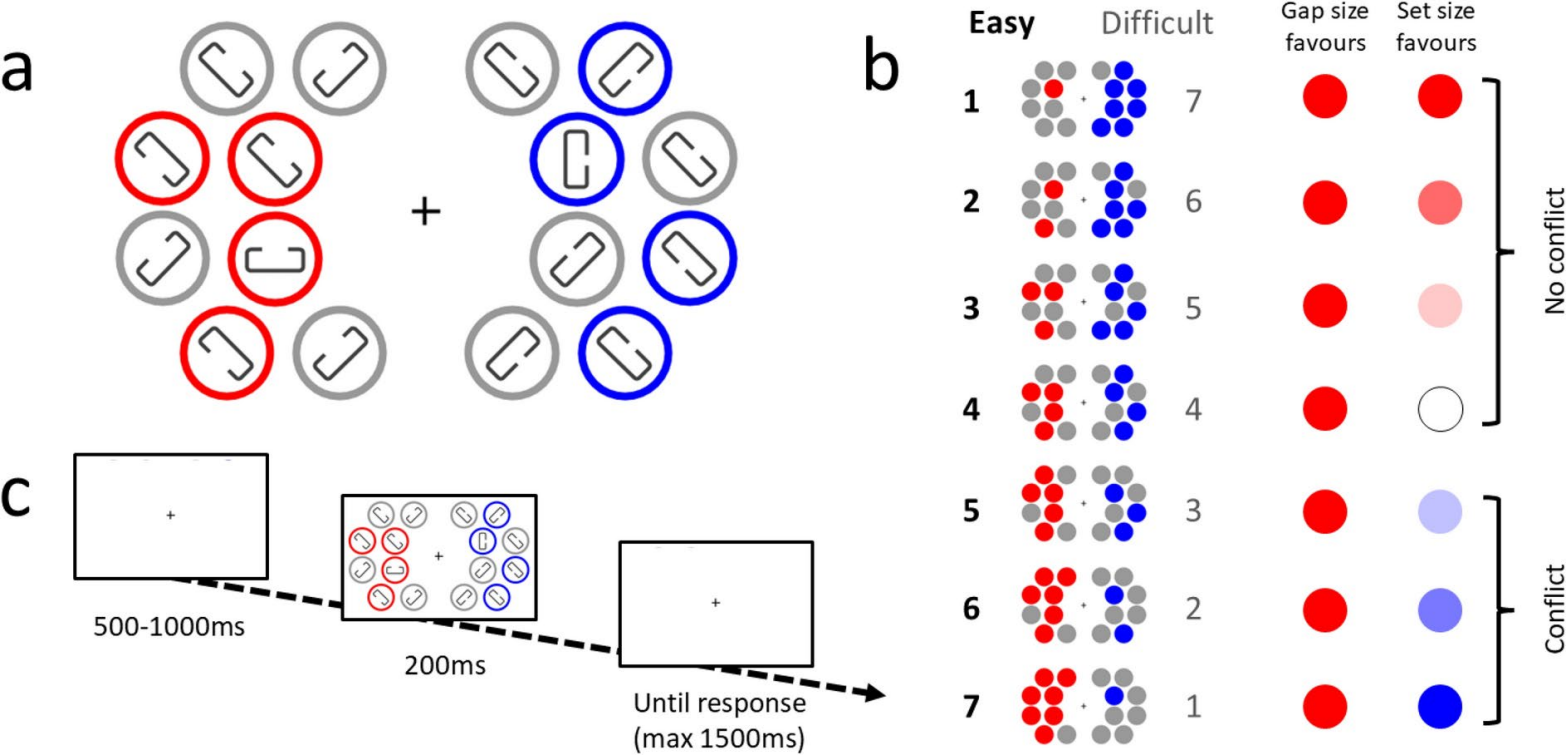
(**A**) Example search display depicting a trial with four easy-coloured rings and four difficult-coloured rings. In this display the easy target is the horizontal rectangle on the left, and the difficult target is the vertical rectangle on the right. Participants must select one of the targets and indicate which side of the rectangle has a gap. Colours mark possible target locations and are associated with each difficulty (counterbalanced across participants). (**B**) A simplified schematic of each condition. Eight coloured rings are presented in each display, but the ratio of easy- to difficult-coloured rings changes across trials (for simplicity, conditions are labelled based on the easy target number). Each colour is restricted to one side of the display for a given trial, and this is counterbalanced across all trials (easy-coloured rings are on the left 50% of the time, and difficult-coloured rings are on the left 50% of the time). On the right, a visual demonstration of how the two factors we manipulate could impact on selection and choice (**C**) An example trial procedure. The initial fixation is on for between 500 and 1000 ms (selected randomly on each trial), followed by the search display for 200 ms, and a fixation which stays on until response (up to a maximum of 1500 ms). Note: displays in the task used a dark grey background, white is shown here for simplification.

**Fig. 2:**
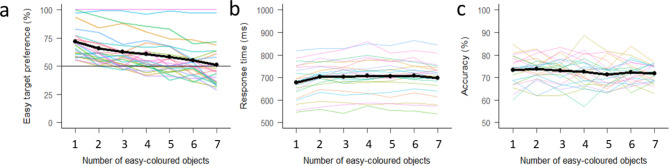
Plots showing the behavioural data across set size condition. (**A**) Easy target preference reflects the percentage of trials in a given condition for which participants chose the easy target. Greater than 50% indicates an average preference in favour of responding to the easy target, below 50% reflects an average preference for responding to the difficult target. (**B**) Response times across set size condition, and (**C**) Response accuracy across set size conditions. In all plots group average data is depicted in black while individual data is shown in coloured lines.

Figure 2 illustrates how preference of the easy target, response time, and accuracy depend on the number of “easy-coloured” objects in the display (cf. Figure 1b). There was a substantial decrease (about 25 percentage points) in the easy target preference with increasing number of easy-coloured objects (Fig. 2a). This is statistically supported by a slope estimate on the log-odds scale of -0.16 [-0.18, -0.14], zero, “no effect”, was outside the 95% highest density interval (HDI). The main effect of preference of the easy target can be seen in its report probability at level four (4 easy vs. 4 difficult). This was estimated at 0.63 [0.49, 0.77], with 95.9% of the posterior density being in the range from 0.5 to 1 that favours the easy target. Both reaction time and accuracy curves are essentially flat (Fig. 2b,c). Statistically, reaction time increases with “easy-coloured" object number, but only with 2.15 ms [0.98, 3.4] per object, zero outside the 95% HDI. Accuracy, decreased by only -0.32 [-0.6, -0.05] percentage points per object, again zero was outside the 95% HDI.

### ERP results

**Fig. 3:**
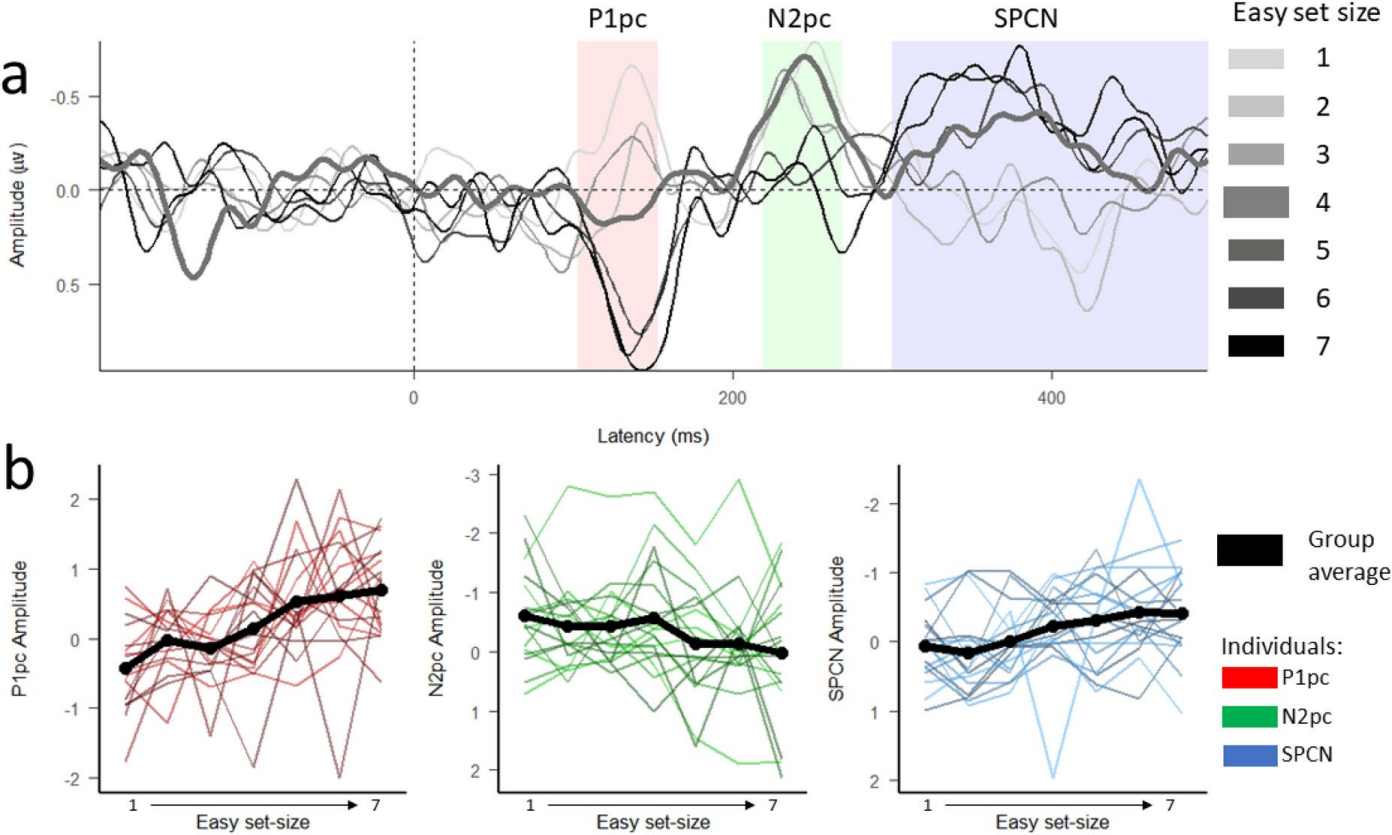
(**A**) Subtracted ERPs time-locked to the onset of a search display showing the lateralized response contralateral to the side of the easy target as a function of easy set size (the 4:4 condition is bolded). P1pc, N2pc and SPCN time windows that were used for calculation of statistics are highlighted in red, green, and blue respectively. (**B**) Each component’s average amplitude is visualised across set size (black line) with individual subject amplitudes depicted in colour. This is plotted for the P1pc component, N2pc component, and SPCN component in red, green, and blue respectively.

All measured ERP components (see Fig. 3b) were modulated by the number of easy-coloured targets (cf. Figure 1b). P1pc amplitude increased as the easy set size increased by 0.19 µV per object [0.13, 0.24], zero, “no effect”, outside the 95% HDI. We also observed a significant effect of set size on N2pc, although in this case N2pc amplitude was attenuated by 0.1 µV per object [0.04, 0.15], zero outside the 95% HDI. Finally, SPCN amplitude increased with easy-coloured set size by − 0.1 µV per object [− 0.14, − 0.06], again, zero outside the 95% HDI.

### Predicting choice behaviour from ERPs

As discussed in "Behavioural results", target choice can in part be predicted based on the set size of the easy-coloured objects. Here we tested whether the ERP components contribute to the prediction of choice as well. As both attentional selection (indexed by the N2pc) as well as visual working memory (indexed by the SPCN) could be associated with the target choice, we tested model variants with different combinations of set size and these ERPs in a model comparison.

**Fig. 4:**
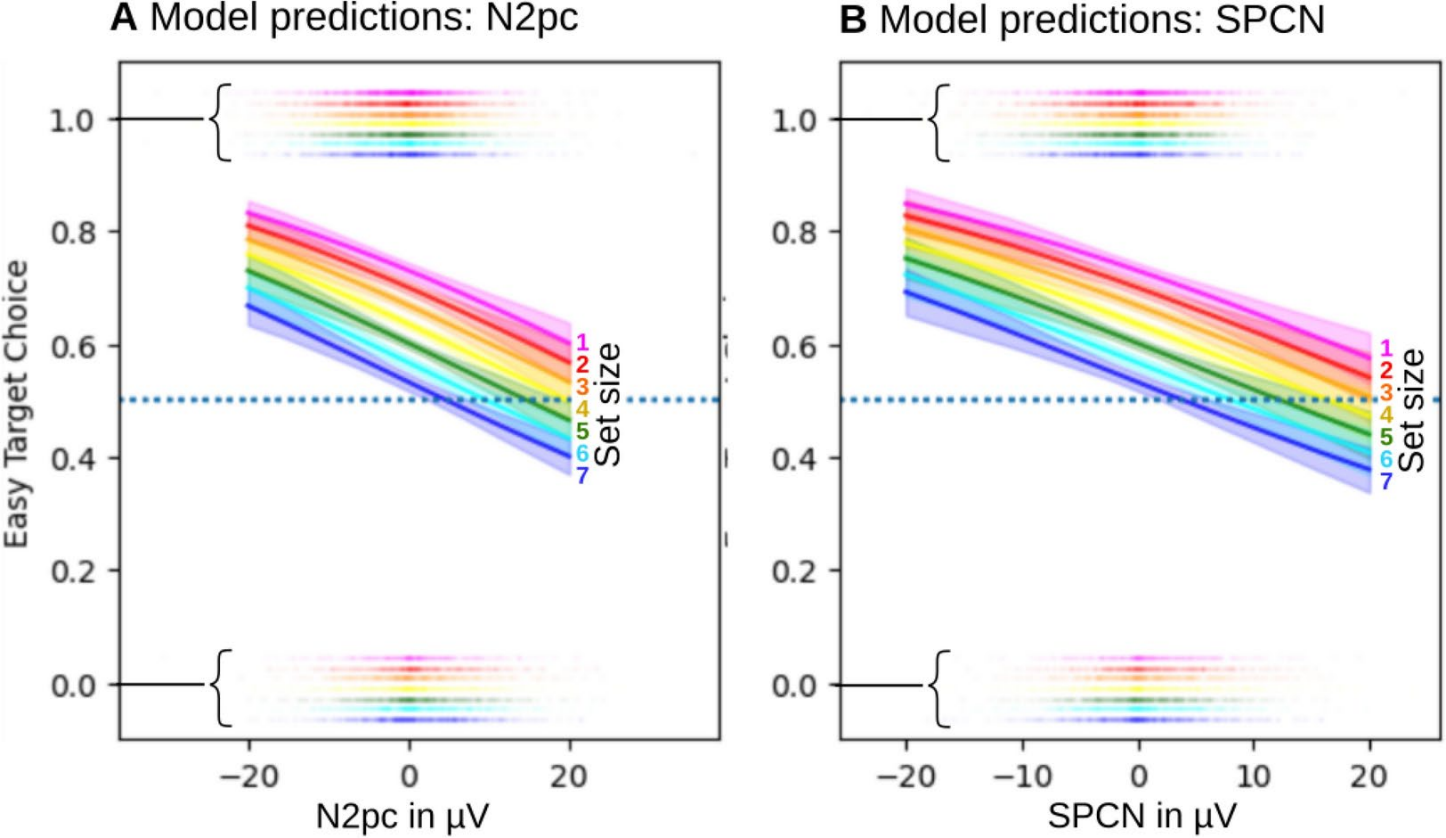
Posterior predictions of easy target choice averaged across participants for each ERP predictor across the different set size conditions (1 easy to 7 easy coloured items). The half-transparent blots at 0 and 1 represent the binary response data. Note the vertical deviations of these blots from 0 and 1 have been introduced to increase visibility of set size differences. The curves represent the mean predicted choice probability and the shaded error bands represent the corresponding 95% highest density interval.

The model that only considers set size of the easy-coloured objects (and no ERP component) can be considered a baseline. As can be seen in Table 1, this "set size only" model reaches rank five in the comparison. Models with just N2pc or SPCN rank below the set size only baseline. All model variants that contain set size plus ERPs as predictors rank higher than the baseline. Models with set size and N2pc (with and without interaction between the two) rank above the models with set size and SPCNs (the versions with interactions, indicated by the "*", are slightly worse than the versions with main effects only). Importantly, the highest-ranking model is the one with main effects of set size and both N2pc and SPCN. More complex models with interactions between N2pc and SPCN suffered from inefficient sampling and reached LOO scores more than three times lower than the worst model listed and were hence omitted from the comparison. As depicted in Fig. 4, the probability of choosing the easy coloured target declines by about 0.6 percentage points per µV increase of the N2pc and by about 0.8 percentage points per µV of the SPCN.

**Table 1:** Different model variants.

Model	Rank	Score	ΔScore	SEΔ	Weight
SetSize + N2pc + SPCN	1	− 7379.92	0	0	90%
SetSize + N2pc	2	− 7407.42	27.50	7.54	5%
SetSize*N2pc	3	− 7408.21	28.29	7.55	0%
SetSize + SPCN	4	− 7415.55	35.63	8.06	5%
SetSize*SPCN	5	− 7416.52	36.61	8.67	0%
SetSize	6	− 7513.66	133.74	16.18	0%
N2pc	7	− 7531.73	151.82	17.27	0%
SPCN	8	− 7559.27	179.36	18.79	0%

“Model” specifies the combined components, where “ + ” refers to models with main effects only. “Rank” from 1 (best) to 8 (worst). “Score” from the leave-one-out cross validation (see section Data Analysis); better models have higher scores. “ΔScore” contains the score difference to the best model. “SEΔ” is the estimated standard error of ΔScore. “Weight” can be loosely interpreted as model probability. Technically it is the weight a model should be given if all the listed models would be jointly applied and averaged to obtain the best prediction.

“*” indicates models with main effects and interactions.

## Discussion

In the present experiment we aimed to evaluate how initial attentional selection impacts on choice in a dual-target task. Our results show that participants can flexibly adapt their target choice to trade-off the ease of a particular target’s gap discrimination, and the search costs involved in finding that target in its current context^2^. While participants showed a strong preference for choosing easily discriminated targets overall, this preference decreased in trials where that target was surrounded by increasing numbers of colour matching distractors. Notably, the trade-off between gap discriminability and set size allowed participants to maintain stable response times and accuracy across conditions. In addition, lateralised ERP components revealed the dynamics of this trade-off at initial stages of attentional selection preceding the response. Early lateralised activity (approximately 100–150ms post-stimulus) showed a highly systematic modulation related to set size that tracked low level stimulus effects. However, in the N2pc time range we observed a modulation of lateralised activity that reflected the interaction of set size and gap discriminability, and this continued in the later SPCN.

Our results were consistent with an N2pc that reflects the initial allocation of attention to items in the contralateral visual field. A large N2pc was elicited by easy targets in displays where the easy target search set was small. This suggests that when there is no conflict between set size and gap discriminability and, therefore, both factors favour the easy target, participants initially allocate their attention to the easy target set. However, in trials where there is a conflict between set size and gap discriminability, the N2pc to easy targets is attenuated (approximating zero). These trials represent a complex set of conditions where participants must determine whether it is more efficient to search through a large set of possible targets to make an easy gap discrimination, or search through a smaller set of possibilities but make a difficult gap discrimination. That we observe an attenuated N2pc in these conditions likely reflects the fact that, as opposed to trials without any conflict, this complex trade-off leads to an inconsistent allocation of initial selection to either side of the display.

When focussing on the SPCN component we again saw a systematic effect of set size on amplitude. In visual search tasks such as ours, SPCN typically reflects the visual short-term memory (VSTM) processes that temporarily maintain target representations while relevant information is extracted and a response is prepared. Our results showed that when the easy target search set was large, it elicited a large SPCN. This suggests that as the conflict between set size and discriminability increased, participants increasingly relied on holding and searching information stored in VSTM to make their choice. Consistent with this, we did not observe a clear SPCN in displays where the easy target search set was small. Given that there is no conflict between set size and discriminability in these trials, participants presumably have an easier time selecting their preferred target and responding without relying on extensive search through VSTM.

Interestingly, and somewhat uniquely, these ERP results demonstrate an inverse relationship between N2pc and SPCN amplitude in our task. In previous visual search tasks that elicit both an N2pc and SPCN, there is typically a positive relationship between their amplitudes^40,41,47,48^. As the N2pc elicited by a target increases, so does the amplitude of its associated SPCN. This makes sense given that in standard visual search tasks where a single pre-defined target is presented on each display, that target item is both most likely to be selected for processing and, therefore, its features are most likely to be stored in VSTM. Consequently, initial work in this area struggled to explicitly dissociate these two components from one another and demonstrate that they represent truly distinct processes^41^. However, our task produced a completely inverted relationship between N2pc amplitude and SPCN amplitude. N2pc is largest in set size conditions where SPCN is smallest or absent, and vice versa. The fact that we have observed such a dissociation in our data may be a consequence of the complex task demands introduced by a dual target search task.

One possibility is that the trade-off required by this task created a dissociation between the processes of target selection and target identification as outlined by Eimer^49^. In this view, N2pc reflects the initial selection of an object, a transient stage of visual processing which produces spatial enhancements at that item’s location. SPCN, on the other hand, typically reflects an identification stage of processing where activation of possible target objects is sustained so that participants can identify features of the stimuli that are response relevant. Importantly, the sustained activity of the identification stage is most useful in conditions where response relevant information could not be extracted from the preceding, transient stage of selection. In our task, when the easy target search set is small, it is typically selected for processing as indexed by the large N2pc. Given that there are relatively few easy-coloured items to search through and the gap size is large, participants may frequently extract response relevant information during selection. In this case, the need for a subsequent identification stage that relies on VSTM maintenance is eliminated and, therefore, no SPCN is observed. At set size four or more, however, participants struggle to reliably extract target information during initial selection, and instead identification is increasingly carried out within VSTM. At these set sizes, we observed a reliable SPCN being elicited by the easy target set.

The possibility that response relevant information is determined either at selection or identification stages, is also consistent with our model predictions for target choice. Model comparisons showed that the best model for predicting choice in our task was a model that included set size condition, N2pc amplitude, and SPCN amplitude. For both N2pc and SPCN, model predictions showed that increased amplitude (more negative) of the component contralateral to a target, predicted increased likelihood that participants would choose that target for their response. Within a set size condition, each of these components accounted for approximately 10% of the variance in target preference. If response relevant information on a given trial can be determined during either the selection or identification stage depending on task demands, then this would explain why each of these components contribute unique information to the prediction of participants’ target choice.

Overall, these results demonstrate not only that participants can flexibly adapt their behaviour to respond to complex trade-offs, but that early attentional processing plays a central role in this ability. The observation that early lateralised components of selection and identification predict choice behaviour is evidence in favour of the hypothesis that attentional processes help to determine which target item participants will ultimately choose, at least within a rapid search and respond task such as this.

An unexpected finding in our data was the highly systematic modulation of early lateralised activity within the time range of a P1pc. While initial work on this component identified it as the product of sensory imbalances in a display, more recent work has shown that it can also reflect intertrial priming or proactive suppression in some cases^13,23,42,43^. In our displays, luminance was balanced across the display using grey filler items. Nevertheless, P1pc showed a robust modulation according to set size such that it was more positive for the side of the display that contained the most coloured targets. Additionally, the amplitude of the P1pc does not appear symmetric about 0 $\mu$ V. Rather, the average P1pc has a trend towards being larger for easy-coloured sets (reaching approximately 1 $\mu$ V contralateral to the largest easy set) than difficult-coloured target (reaching approximately 0.5 $\mu$ V contralateral to the largest difficult set). This suggests that rather than being a reflection of pure sensory energy, P1pc incorporates the relevance of the possible stimuli on screen favouring coloured over grey stimuli, as well as some element of target preference, favouring easy over difficult. However, the functional relevance of P1pc in our data remains unclear.

An alternate interpretation of this early component in our task is that is reflects N1pc, an early lateralised negativity, however, the bilateral nature of our displays makes it difficult to disentangle from early lateralised positivities. The N1pc is a lateralised negativity elicited contralateral to salient targets in a visual search display.^42^ The N1pc typically peaks withing a 100-150 ms post-stimulus time range and reflects a more automatic, initial spatial bias toward a stimulus than the later N2pc. In our paradigm, each trial has two targets, one in each visual field. This makes it difficult, in principle, to determine whether the early lateralised component in our results reflects a contralateral positivity (P1pc) elicited by the side of the display with more coloured items, or a contralateral negativity (N1pc) elicited by the opposite side of the display because of its smaller set size. While Verleger and colleagues distinguished P1pc and N1pc based on timing, suggesting that P1pc was defined by an earlier peak between 60-100 ms post stimulus, subsequent work has identified P1pc components ranging anywhere from 70 to 210 ms post stimulus.^50–55^.

Early work on N1pc showed that it was associated with the initial orienting of attention to a salient target stimulus.^56,57^ More recently in contextual cuing paradigms, where previous experience of a display’s layout guides attention to the target location, N1pc has also been shown to be elicited by the learned target location.^58–60^ Importantly, these studies have shown that N1pc continues to be elicited by the learned location, even on trials where the target itself is unexpectedly moved to the opposite hemifield. Contextual cuing is an implicit process^61,62^ and, therefore, these findings in conjunction with the earlier work on target saliency imply that N1pc may index the initial, automatic guiding of attention to a likely target. If the early lateralised activity in our task is an N1pc then this would imply that initial, automatic guidance of attention in our task is toward the side with the smaller set size. This could imply that the smaller set size operates as a pop-out, attracting initial automatic orienting. However, optimal decision-making about which target to respond to requires an integration of this initial set size effect along with participant information about which target is easier to respond to. The integration of these two factors is seen in the N2pc time window which also, therefore, predicts the target choice that participants ultimately make.

## Conclusion

In this study we expand on previous work investigating how people make complex trade-offs in dual-target tasks by measuring the neural processes that precede target choice. We used a dual-target search task that allows for the measurement of lateralised ERP components as indices of attentional processing, to understand whether and how attention contributes to the trade-offs required by conflicting task demands. Our results confirm previous work suggesting that participants can flexibly adapt their choice behaviour from trial to trial to maintain speed and accuracy. However, this behaviour is underpinned by a complex interplay between initial attentional selection and target identification within VSTM. Attentional selection as indexed by the N2pc, maps onto the presence or absence of conflict between task demands, whereas VSTM is recruited as initial selection becomes insufficient for identifying response-relevant information. Bayesian multi-level modelling revealed that N2pc amplitude and SPCN amplitude are useful predictors of a person’s target choice. These results are consistent with lateralised attentional components that reflect selection, and identification within VSTM, and also suggest that selection and identification stages help to determine the target choice that a participant will make. Additionally, our data highlight the rich information that can be extracted from complex visual search tasks where participants flexibly, and freely adapt their choice strategies based on competing task demands.

## Supplementary Information

Supplementary Information.

## Data Availability

The datasets analysed in the current study will be available from zenodo, and by request from the corresponding author.
