# Author Correction: Complex trade-offs in a dual-target visual search task are indexed by lateralised ERP components

**DOI:** 10.1038/s41598-024-78263-z

**Published:** 2024-11-06

**Authors:** Dion T. Henare, Jan Tünnermann, Ilja Wagner, Alexander C. Schütz, Anna Schubö

**Affiliations:** 1 Auckland University of Technology, 90 Akoranga Drive, Auckland, 0627 New Zealand; 2 Philipps-University of Marburg, Marburg, Germany; 3 Justus Liebig University, Giessen, Germany

Correction to: *Scientific Reports* 10.1038/s41598-024-72811-3, published online 01 October 2024

The Funding section in the original version of this Article was omitted. The Funding section now reads:

“ACS, AS, DTH and IW were funded by the Deutsche Forschungsgemeinschaft (DFG, German Research Foundation) – project number 222641018 – SFB/TRR 135 TP B2 and TP B3. ACS AS, and JT received funding from the Excellence Program of the Hessian Ministry of Higher Education, Science, Research and Art ("The Adaptive Mind”).”

The original Article has been corrected.

