# Supplementary material for: Complex trade-offs in a dual-target visual search task are indexed by lateralised ERP components
Source: Sci Rep. 2024 Oct 1;14:22839. doi: 10.1038/s41598-024-72811-3 (PMC11448495; doi:10.1038/s41598-024-72811-3)
Supplement: Supplementary file 1 — Supplementary Information. [file 41598_2024_72811_MOESM1_ESM.docx]

**Supplementary information**

**Complex trade-offs in a dual-target visual search task are indexed by lateralised ERP components.**
**Dion T. Henare^1*^, Jan Tünnermann^2^, Ilja Wagner^3^, Alexander C. Schütz^2^, & Anna Schubö^2^**
^1^ Auckland University of Technology, New Zealand, ^2^ Philipps-University of Marburg, Germany, ^3^Justus Liebig University Giessen, Germany

**S1. Static training**

The goal of the static training was to provide participants with some experience in the discrimination task and find a starting value for further adjustment of the difficulty in the staircase training described below. Both trainings were conducted on a 32" Display++ (Cambridge Research) at a resolution of 1920 x 1080 pixels, the distance between screen and participants was 90 cm. A chin rest was used. Similar to the main experiment, six items were presented to each side of the fixation mark as two vertical columns with three items on each side (x-distance inner rows: 174 pixels x-distance outer rows: 294 pixels; y-distances: -120, 0, and 120 pixels). A random jitter of 0 to 25 pixels was applied to the items. The items were rings (92 x 92 pixels) with inner tilted or straight Cs as in the main experiment. The items differed from the main experiment only in the ring colour, which was always grey for all items. As in the main experiment, there was one target with a horizontal or vertical C on each side of the fixation mark. The Cs in the distractors were tilted by 40°. Different from the main experiment (and the staircase training described below), both targets' Cs always had the same gap size, initially 8 pixels. The presentation sequence was as follows: The central fixation mark was shown alone for 700 to 1200 ms and then search display was presented contingent on central fixation (controlled with an EyeLink 1000+ plus eye tracker; SR Research). The items were visible for 200 ms, followed by a blank screen. Participants reported the gap location of their chosen target as in the main experiment.

Participants performed in blocks of 60 trials. After each block, the average proportion of correct target detections for the first, middle, and last 20 trials of a block were shown to the experimenter. The experimenter decided to continue the training with more blocks or to conclude the static training. When continuing the training, the experimenter decided whether to increase of decrease the gap size (of both targets' Cs), based on how well participants performed. The goal was to reach stable performance around 80 % of correct reports. When this was reached, the static training was concluded. On average participants conducted five blocks. Their final gap size was used to inform the starting point for the staircase training.

**S2. Staircase training**

The goal of the staircase training was to further familiarize the participants with the task and adjust the easy and difficult gap sizes to the individual performance. The setup, stimuli and presentation sequence were as in the static training described above. However, the goal of the staircase training was to find two gap sizes of different difficulty (one easy, one difficult) individually for each participant. To this end, two QUEST ^1^ staircase procedures were und in parallel for the easy and difficult gap sizes. The starting gap size for both targets was the one determined in the static training and the target performance was a proportion of 0.7 correct for the difficult and 0.9 for the target. Participant performed again on average 5 blocks. The target proportions correct were not always achieved and some sessions had to be terminated due to running out of time. But as long as the difficult gap size was estimate more difficult than the easy gap size, the participants could perform the main task and their individually tuned gap sizes were used.

1. Watson, A. B., & Pelli, D. G. (1983). QUEST: A Bayesian adaptive psychometric method. *Perception & psychophysics*, *33*(2), 113-120.
